# From sky to sow: assessing the impact of drone-enabled semen delivery on pig fertility outcomes in rural Rwanda

**DOI:** 10.3389/fvets.2026.1791617

**Published:** 2026-03-23

**Authors:** Jean Marie Providence Manikuzwe, Pedro Kremer, Maria Jose Ospina Fadul

**Affiliations:** 1 Zipline Rwanda Ltd., Kigali, Rwanda; 2 Zipline International Inc., South San Francisco, CA, United States

**Keywords:** aerial logistics, artificial insemination, Community Animal Health Workers (CAHWs), drones, pig semen delivery, Rwanda Agriculture and Animal Resources Development Board (RAB)

## Abstract

**Introduction:**

Improving livestock productivity is vital for food security and rural incomes in Rwanda. Pig farming offers rapid income potential but is constrained by limited access to timely, high-quality artificial insemination (AI) services. To address this, the Rwanda Agriculture and Animal Resources Development Board (RAB), Zipline, and USAID’s Orora Wihaze project piloted an aerial logistics model to distribute pig semen via drones, combined with training and awareness activities. This study evaluates the program’s impact on AI outcomes among veterinarians, Community Animal Health Workers (CAHWs), and farmers across eight districts.

**Methods:**

A pre–post design with repeated cross-sectional surveys was conducted over 6 months. Baseline and endline surveys included veterinarians (*n* = 257/221), CAHWs (*n* = 310/240), and farmers (*n* = 233). Data covered AI success rates, procedural challenges, and economic outcomes. Regression models identified predictors of AI success, and an input–output analysis estimated broader economic impact.

**Results:**

AI performance improved significantly, especially among CAHWs, whose success rates rose from 48.8 to 74.8% (*p* < 0.05). A binomial GLM confirmed higher odds of success at endline (OR = 1.51, 95% CI: 1.23–1.87; *p* = 0.00012). Technical and procedural failures declined, and cold chain and expired semen issues decreased, indicating improved skills and logistics. The program resulted in 4,489 inseminations and 28,553 piglets, with 4,893 attributable to program-driven gains. Farmers earned $320,075, of which 17.1% stemmed from the intervention. With an implementation cost of $191,149, the direct ROI was 67.5%. Including indirect and induced effects, total impact reached $2.3 million.

**Discussion:**

Drone-based semen delivery, paired with capacity-building, can improve reproductive outcomes, enhance last-mile delivery, and strengthen rural economies.

## 1 Introduction

Aerial logistics involves using drones to efficiently transport goods, especially sensitive biological materials like semen for artificial insemination, to remote areas—reducing reliance on ground transport and ensuring timely, cold chain-compliant delivery in agriculture and animal health. However, achieving high fertility rates in pig production is often constrained by limited access to timely and viable artificial insemination (AI) services, particularly in remote and underserved areas.

Pig semen is a highly temperature sensitive biological product with a limited shelf life, necessitating stringent cold chain management and rapid delivery to maintain fertility potential. Traditional distribution systems frequently encounter logistical challenges, such as delivery delays, temperature inconsistencies, inadequate last mile coverage, and insufficient knowledge and awareness among key actors in the pig AI service ecosystem, including veterinarians, Community Animal Health Workers (CAHWs), and farmers. These barriers collectively contribute to reducing artificial insemination success rates and hinder the broader adoption of AI technologies among smallholder pig farmers. Rwanda’s pig production system is characterized by fragmented value chains, governance constraints, and sanitary vulnerabilities that further complicate access to reliable reproductive inputs (1).

In response to these challenges, Zipline, in collaboration with the Rwanda Agriculture and Animal Resources Development Board (RAB) and USAID Feed the Future’s Orora Wihaze project implemented an innovative aerial logistics model using drone technology. Over a 12 month period, the approach’s core objectives included improving the delivery and quality of AI services, building the capacity of key stakeholders, and boosting awareness and demand for AI technologies across eight targeted districts: Ngoma, Kayonza, Gakenke, Burera, Ngororero, Rutsiro, Nyamagabe, and Nyamasheke.

To achieve these goals, the project leveraged Zipline’s aerial logistics built around centralized storage and drone-based distribution, operating from two strategically located distribution centers to ensure the rapid, temperature-controlled delivery of pig semen. These centers manage inventory and dispatch semen doses via autonomous drones, enabling same-day delivery to remote AI service providers while maintaining strict cold chain requirements. In addition, the project implemented a range of complementary interventions, including training for AI centers, pig multipliers, and CAHWs; Pig Business Linkage Meetings; AI awareness campaigns; and the provision of semen storage equipment to AI centers. The initiative aimed to enhance service delivery efficiency, strengthen stakeholder engagement, and support the effective integration of technology into Rwanda’s livestock production systems.

This study presents the findings from the evaluation of the aerial logistics model for swine AI service delivery. It assesses changes in service delivery performance, AI success rates, and the perceptions of veterinarians, CAHWs, and farmers regarding the quality and effectiveness of drone-based swine semen distribution. The results are also used to estimate the direct, indirect, and induced economic impacts of the program. Together, these findings offer insights into the feasibility, impact, and potential for scaling technology driven livestock interventions in Rwanda and comparable settings.

## 2 Materials and methods

### 2.1 Study design and population

This study employed an observational design. For veterinarians and CAHWs, a prospective cohort was used. For served pig farmers, a single survey was conducted at the endline. Surveys captured both quantitative and qualitative data, including procedural success rates, cold chain handling, and piglet output to measure impact, statistical models compared baseline and endline performance, while controlling for individual and district-level characteristics.

**Figure 1:**
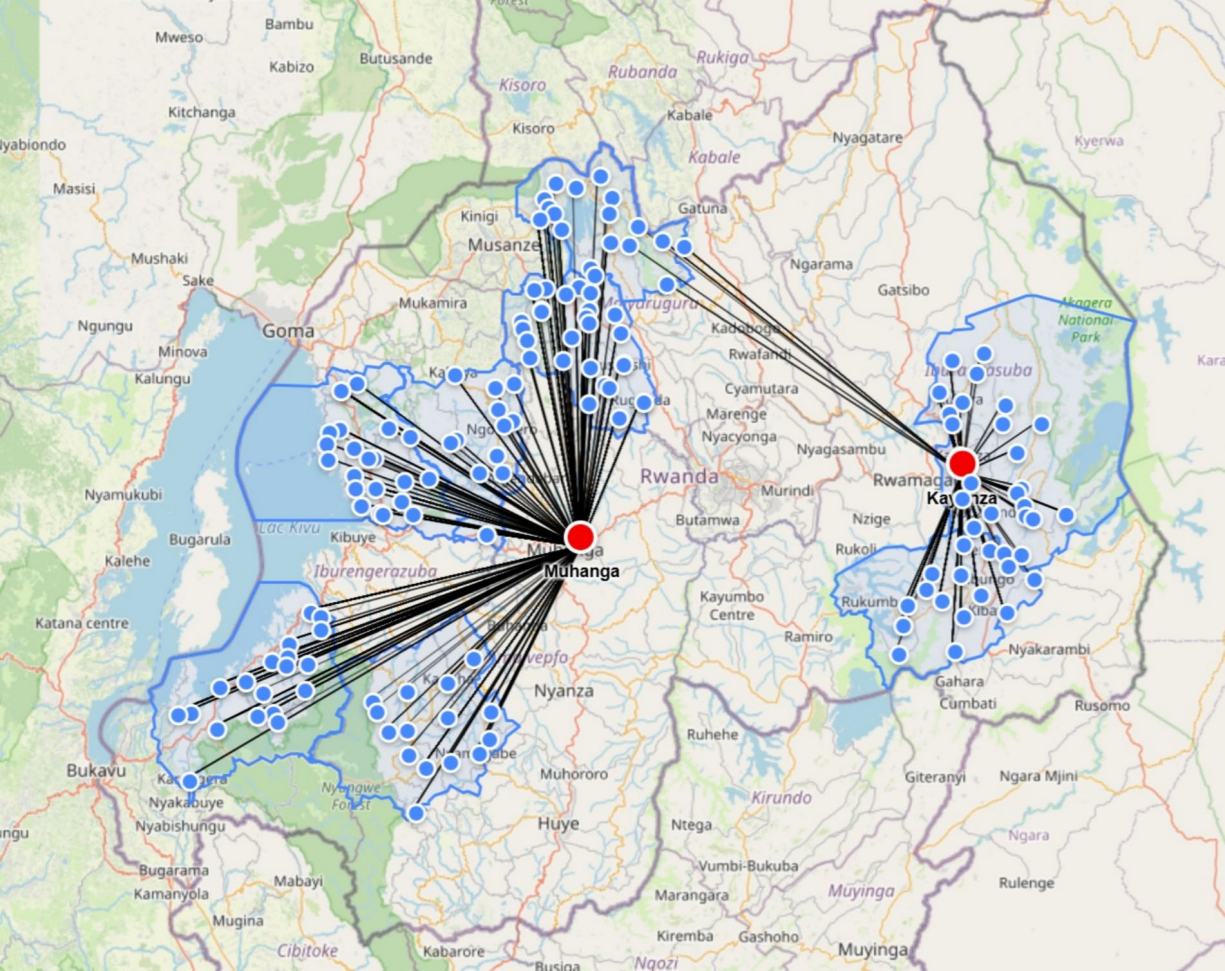
Swine semen deliveries from two distribution centers. Reproduced with permission.

This study was conducted across eight districts in Rwanda: Ngoma, Kayonza, Gakenke, Burera, Ngororero, Rutsiro, Nyamagabe, and Nyamasheke. These locations represent the targeted intervention areas of the Orora Wihaze Project (Figure 1).

The study population consisted of three primary stakeholder groups directly involved in the delivery and utilization of swine AI service. For veterinarians, a total of 257 participants were surveyed at baseline, and 221 at endline. For CAHWs, 310 respondents were surveyed at baseline and 240 at endline. A representative sample of 233 farmers who had received swine semen between October 2023 and June 2024 was included.

The study was designed to follow the same cohort of veterinarians and CAHWs over time. Differences in baseline and endline sample sizes reflect attrition due to routine programmatic factors rather than selective exclusion. For veterinarians, 36 participants (14.0%) were not re-interviewed at endline, while for CAHWs 70 participants (22.6%) were lost to follow-up. Attrition occurred primarily due to employment changes, reassignment to non-AI responsibilities, or inability to reach participants after repeated contact attempts. No participants were excluded based on reported AI outcomes.

The sample of 233 farmers at endline was drawn from the administrative registry of all farmers who received at least one swine semen dose between October 2023 and June 2024 across the eight intervention districts. A sampling frame was constructed from this registry, and farmers were randomly selected and contacted by phone. Inclusion criteria required documented receipt of semen during the intervention period and availability of a valid contact number. No exclusion criteria were applied based on reported insemination outcomes.

The intervention leveraged a tiered service model in which veterinarians maintained supervisory and quality assurance roles, while CAHWs provided decentralized frontline service delivery to farmers. In Rwanda, veterinarians are primarily responsible for ordering and administering the AI, while CAHWs play a complementary role by mobilizing farmers and promoting good husbandry practices. The majority of endline participants had direct experience with Zipline-delivered swine semen, positioning them to effectively assess the intervention’s technical performance and community-level impact. This composition enabled a comprehensive evaluation of the aerial logistics model in terms of service delivery, adoption, and stakeholder engagement across the targeted rural districts.

### 2.2 Semen procurement, preparation, and handling

Swine semen doses were sourced from two approved channels: (1) the Rwanda Agriculture and Animal Resources Development Board (RAB) boar stud facility, and (2) licensed private breeding centers operating under national veterinary regulatory standards.

All semen consisted of extended fresh semen prepared under controlled laboratory conditions. Following collection from approved boar studs, ejaculates underwent standard quality control procedures, including evaluation of sperm motility and concentration using microscopy. Only ejaculates meeting predefined minimum quality thresholds were processed for artificial insemination.

Semen was diluted using commercially available swine semen extenders formulated to maintain sperm viability during short-term storage. Dilution was performed to achieve standardized AI dose volumes consistent with national artificial insemination guidelines.

Prepared doses were packaged in sterile containers and stored under controlled refrigeration at approximately 17 °C, the recommended temperature range for extended fresh swine semen. Storage duration prior to dispatch followed national guidelines to preserve viability and minimize bacterial proliferation.

For aerial distribution, semen doses were transported from centralized storage facilities in temperature-controlled containers designed to maintain recommended thermal conditions throughout transit. Cold chain integrity was monitored through standardized handling protocols to minimize temperature excursions prior to insemination.

Swine semen used in this study consisted of extended fresh semen rather than cryogenically frozen semen. Unlike bovine semen, which is commonly stored in liquid nitrogen at −196 °C, swine semen is typically maintained at controlled refrigeration temperatures around 17 °C to preserve sperm viability while avoiding cold shock.

Cold chain integrity during aerial transit was maintained through a combination of logistical and handling mechanisms. Semen was stored in temperature-controlled refrigeration units at centralized distribution centers prior to dispatch. Each shipment was packaged in insulated transport containers designed to maintain internal temperatures within the recommended range during flight and short-term handling. Finally, the relatively short transit times enabled by drone delivery reduced overall exposure to environmental temperature fluctuations compared to conventional ground transport. Upon arrival at the designated delivery location, semen doses were promptly transferred to veterinarians or CAHWs for appropriate storage or immediate use.

### 2.3 Statistical analysis

Descriptive statistics were used to summarize demographic characteristics and outcomes across all groups. For veterinarians and CAHWs, baseline-to-endline changes in continuous outcomes were assessed using paired sample t-tests, and changes in categorical outcomes were evaluated using McNemar’s test, appropriate for repeated measures within individuals. For farmers, descriptive analyses summarized sow characteristics (age, breed, parity), AI success rates, and average piglet counts per litter.

The primary focus of inferential analysis was on AI success rates among CAHWs, who served as frontline AI providers with direct interaction with farmers. Due to their close proximity to smallholders and their routine involvement in follow-up visits, CAHWs were considered the most reliable source of outcome data on insemination success. To assess factors associated with AI performance, we conducted two regression models using pooled baseline and endline CAHW data. First, an ordinary least squares (OLS) regression was used with the self-reported AI success rate—defined as the proportion of successful inseminations in the past 30 days—as the dependent variable. Independent variables included respondent sex, age, years of experience as a CAHW, years of AI knowledge, district, and survey round (baseline or endline). Second, a binomial generalized linear model (GLM) modeled the number of successful versus unsuccessful procedures per CAHW, using the same covariates and including district-level fixed effects to account for geographic variation in implementation context. For veterinarians, although AI success rates were also collected, inferential analysis focused on perceived changes in AI-related procedural challenges—such as expired semen, cold chain failures, and heat detection difficulties—between baseline and endline. This emphasis reflected veterinarians’ supervisory and coordination roles rather than their direct involvement in follow-up with farmers. Among farmers, we used logistic regression to model the odds of reported AI success (yes/no) based on sow characteristics such as breed, age, and pregnancy history, controlling for district fixed effects. All analyses were conducted using Stata version 17 (StataCorp, College Station, TX), with statistical significance defined as *p* < 0.05.

### 2.4 Economic analysis

To estimate how improvements in swine insemination success rates translate into economic gains, we modeled the expected increase in net piglet production by applying the incremental success rate, the full-term pregnancy rate measured in the endline survey, and the average litter size for AI-induced full-term pregnancies from literature. We then multiplied this figure by the number of doses distributed through the program. Finally, we estimated the additional income generated by multiplying the resulting number of piglets by their average market value in Rwanda.

To assess how this direct increase in farmer income translates into benefits for the broader economy, we employ multiplier analysis within an input–output (I-O) modeling framework. These models quantify how changes in one sector affect the entire economy by capturing three distinct effects: direct, indirect, and induced. Each of these effects corresponds to a specific type of multiplier. The direct effect (captured by the base output) represents the immediate increase in production and income within pig farming.

The indirect effect, measured by the Type I multiplier, reflects how increased production in the swine sector stimulates demand in upstream supply chains (e.g., feed, veterinary services, construction). This demand stimulates upstream economic activity: feed crop producers and commercial feed manufacturers benefit from higher sales of maize, soybeans, and mixed feed; carpenters and suppliers of building materials see increased business as farmers invest in improved pig housing and equipment; and veterinarians, animal health technicians, and artificial insemination service providers experience heightened demand (2).

The induced effect, captured by the Type II multiplier, accounts for how increased household income from swine production is spent on goods and services, further stimulating economic activity in other sectors. Induced effects are particularly significant in rural, smallholder contexts. As pig farmers earn higher incomes, they tend to spend a substantial portion of that income on locally available goods and services. Increased income also facilitates greater investments in education and health: families are more likely to afford school fees, uniforms, and supplies, and to participate in Rwanda’s community-based health insurance program or seek formal medical care. These expenditures generate additional employment and income for shopkeepers, transporters, teachers, and healthcare workers. Because this income is largely spent and re-spent within the local economy, the resulting induced effect (captured by the Type II multiplier) can be substantial.

Rwanda has developed the analytical tools necessary for such assessments over the past decade. The country’s first two I-O tables were developed for the years 2006 and 2011, allowing for the calculation of output, income, and employment multipliers across sectors (3). This study uses the most recent available I-O estimates for Rwanda from (4).

Agriculture and livestock sectors in Rwanda exhibit distinct multiplier characteristics due to the highly localized nature of rural production systems. Smallholder agriculture often features limited inter-industry linkages, as producers typically consume or sell outputs with minimal processing or input from other sectors. Consequently, the Type I output multipliers for agricultural subsectors are relatively low. Specifically, the multiplier for “Livestock and livestock products” is about 1.16, meaning that every additional RWF of final demand generates just 1.16 RWF in total output, including both direct and indirect effects (4).

However, when the induced effect is included (captured by the Type II multiplier) the overall economic significance of agriculture increases markedly. Rural agricultural activities generate labor income that is predominantly spent on goods and services, thereby stimulating demand across other sectors. As a result, the Type II multipliers, which combine direct, indirect, and induced effects, are substantially higher for agricultural activities. According to the I-O analysis, once household spending is incorporated, both livestock and crop production rank among the top contributors to overall economic output. The livestock sector, for example, has a Type II output multiplier of approximately 7.2 (4), implying that each additional RWF of final demand for livestock products ultimately generates about 7 RWF in total economic output. This ranks second among all sectors and highlights a key insight: agricultural income in Rwanda tends to recirculate within local economies rather than leaking out, leading to strong induced effects and high overall multipliers, even when direct supply chain linkages are limited.

## 3 Results

### 3.1 Population and descriptive statistics

#### 3.1.1 Veterinarians

A total of 257 veterinarians participated in the baseline survey and 221 at endline. Respondents had an average age of 33.6 years, with 81% identifying as male, and a mean professional experience of 8.8 years. District-level participation was consistent across both time points, with the majority of veterinarians located in Gakenke, Nyamagabe, Nyamasheke, Kayonza, and Ngoma. Baseline demographic characteristics did not differ significantly between participants retained at endline and those lost to follow-up.

The mean number of artificial insemination (AI) procedures performed increased from 3.59 at baseline to 5.34 at endline. Reported AI success rates remained stable (74.5 to 74.8%) with reduced variability over time, and a weak positive correlation was observed between age and success rates (*r* = 0.16).

Common challenges included heat detection difficulties (rising from 50.4 to 53.9%) and an increase in expired semen cases (7.1 to 11.8%). Significantly, technical difficulties or procedural mistakes saw a sharp and statistically significant decline from 4.9 to 0.4%, suggesting an improvement in the technical execution of artificial insemination procedures.

#### 3.1.2 Community Animal Health Workers

A total of 310 Community Animal Health Workers (CAHWs) were surveyed at baseline, and 240 at endline. The average respondent age was 39 years, with 66% identifying as male. Geographic distribution was consistent across both time points. On average, respondents reported 3.5 years of professional experience, with a median of 2 years. Most had limited exposure to artificial insemination (AI), with 210 CAHWs reporting less than 2 years of AI experience at baseline and an average of 1.39 years of AI knowledge.

The mean AI success rate improved significantly from 48.8% at baseline to 74.8% at endline, a 53% relative increase that was statistically significant (T-test for paired samples *p*-value <0.05).

Reported reasons for AI failure varied between time points. Heat detection difficulties (23.8 to 27.8%) and animal health issues (22.1 to 24.0%) increased slightly, while failures due to expired semen (11.8 to 8.0%) and interrupted cold chains (10.5 to 5.0%) showed statistically significant declines, indicating improvements in product handling and delivery. A statistically significant reduction was also observed in technical or procedural mistakes, dropping from 6.8 to 2.0%. These trends suggest both improved technical competency and the positive effects of targeted interventions during the program period.

To explore factors associated with higher AI success rates, we conducted an ordinary least squares (OLS) regression using combined baseline and endline data. The dependent variable was the AI success rate, calculated as the proportion of successful inseminations out of total procedures conducted in the past 30 days. The model included sex, age, years of experience working as a CAHW, years of knowledge about AI, district, and survey round as predictors.

Individual-level characteristics—including sex, age, years working as a CAHW, and years of AI knowledge were not significantly associated with AI success. However, district-level effects were pronounced. CAHWs in Nyamagabe, Nyamasheke, and Rutsiro had statistically significant higher AI success rates.

A binomial generalized linear model using the number of successful and unsuccessful inseminations reported by each CAHW found that the odds of a successful AI procedure were significantly higher at the endline. CAHWs surveyed at the endline were 51% more likely to report successful procedures (OR = 1.51; 95% CI: 1.23–1.87; *p* = 0.00012).

#### 3.1.3 Farmers

A total of 233 farmers who received swine semen in January 2024 were surveyed. Participants were geographically distributed across the eight districts, with the highest representation in Gakenke (28.5%), Nyamasheke (20.7%), Burera (11.2%), and Nyamagabe (10.8%). Sector, cell, and village level data showed wide diversity, with no single locality dominating the sample. Baseline demographic characteristics did not differ significantly between participants retained at endline and those lost to follow-up.

The sows in the study had a mean age of 14.88 months (median: 12 months), ranging from 3 to 96 months. The average number of previous pregnancies was 0.70, and most sows (approximately 80%) were undergoing artificial insemination (AI) for the first time. Just over half (55.2%) of the sows had never been pregnant prior to the reported AI procedure.

The mean piglet count from the most recent pregnancy was 7.99, with a range of 1 to 16 piglets. Reported AI success was 63.1, and 79.6% of sows carried their pregnancies to full term, suggesting a strong overall outcome in terms of fertility and delivery success.

Extrapolating the total number of doses delivered (over 5,600) during the project, and a mean success rate of 72.8% for a mean number of live piglets of 8, the initiative resulted in 4,075 piglets.

To explore predictors of AI success, we conducted a series of logistic regression models using farmer-reported success (yes/no) as the outcome. The three districts that were independently associated with success from the CAHWs perspective were not significantly associated with success. Also, pregnancy history did not show a significant predicting capacity. We found that sows identified as large white had significantly lower odds of reported success compared to Landrace sows (OR = 0.24, 95% CI: 0.06–0.98, *p* = 0.046; Table 1).

**Table 1:** Veterinarians, CAHWs, and Farmers AI procedures.

Indicator	Vets baseline	Vets endline	CAHWs baseline	CAHWs endline	Farmers endline
Avg. AI procedures per month	3.6*	5.3*	3.2	4.3	
Interrupted cold chain (%)	7.5	8	6.8*	4.2*	
Expired semen (%)	8.6*	11.5*	11.8*	8.4*	
Heat detection difficulty (%)	5	5.4	3.9	4.3	
Animal health issues (Previous) (%)	15.8*	8.8*	25.1*	14.1*	
AI success rate (%)	74.5	74.8	48.8*	74.7*	63.1
Mean piglet count					8
Avg. sow age (months)					14.9
First-time AI (%)					80
Full-term pregnancy rate (%)					79.6

*Baseline/Endline differences are statistically significant.

### 3.2 Economic analysis results

#### 3.2.1 Base case

Table 2 demonstrates the economic impact of the swine insemination program. The program demonstrated strong overall impact, with a marked increase in success rates and resulting farm-level income. The baseline success rate for AI procedures was 48.8%, while the observed endline success rate rose to 74.8%. When adjusting for baseline characteristics using a binomial GLM, the model-predicted success rate under the treatment was 59.0%, corresponding to an odds ratio of 1.51. This suggests that while some of the improvement is explained by broader system-wide changes, a significant portion, 10.2 percentage points, is directly attributable to the aerial delivery model deployed through Zipline’s logistics network.

**Table 2:** Base case results using GLM OR + literature-sourced price per piglet.

Parameter	Value	Source
Baseline success rate	48.80%	CAHWs Baseline survey
Odds ratio success rate at endline	1.51	Findings from binomial GLM for CAHWs
Endline success rate	74.80%	CAHWs Endline survey
Model-adjusted success rate under treatment	59.00%	Calculated based on parameters above
Incremental success rate (AI)	10.20%	Calculated based on parameters above
Full term pregnancy rate	79.60%	Full term pregnancy rate - edline report
Doses of swine semen distributed	7,540	Operational data from Zipline
Total farmers served	3,421	Operational data from Zipline
Average piglets per successful insemination	7.99	(5)
Mean price per weaned piglet	$11.21	Shyaka 2022 brought to 2024 USD
Total successful AI	4,489	Calculated based on parameters above
Total FT pregnancy	3,574	Calculated based on parameters above
Total piglets	28,553	Calculated based on parameters above
Total income	$320,074.79	Calculated based on parameters above
Average income per farmer	$93.56	Calculated based on parameters above
Incremental successful AI	769	Calculated based on parameters above
Incremental FT pregnancy	612	Calculated based on parameters above
Incremental piglets	4,893	Calculated based on parameters above
Incremental income, total	$54,850	Calculated based on parameters above
Incremental income, per farmer	$16.03	Calculated based on parameters above
Multiplier I (indirect effects)	1.16	(4)
Multiplier II (induced effects)	7.20	(4)
Program economic impact, direct	$320,075	Calculated based on parameters above
Program economic impact, indirect	$51,212	Calculated based on parameters above
Program economic impact, induced	$1,933,252	Calculated based on parameters above
Incremental economic impact, direct	$54,850	Calculated based on parameters above
Incremental economic impact, indirect	$8,776	Calculated based on parameters above
Incremental economic impact, induced	$331,294	Calculated based on parameters above
Program economic impact, direct + indirect + induced	$2,304,538	Calculated based on parameters above
Incremental economic impact, direct + indirect + induced	$394,919	Calculated based on parameters above

In practical terms, the program resulted in 4,489 successful inseminations, 3,574 full-term pregnancies, and an estimated 28,553 piglets born, generating $320,075 in direct income for 3,421 farmers. When accounting for indirect and induced effects using multipliers from Al-Ali (4), the total program economic impact reaches $2.30 million, reflecting not only direct revenue gains for farmers but also broader ripple effects throughout the local economy.

While the total economic impact is substantial, the incremental effect of aerial logistics is also notable. Of the total program impact, $394,919 is attributable specifically to aerial delivery, including $54,850 in direct farmer income and over $331,000 in induced benefits. At the individual level, the program resulted in an average income of $93.56 per farmer, of which approximately 17% is directly linked to the aerial logistics model.

#### 3.2.2 Program costs

The total implementation cost of the swine artificial insemination program was $191,149, covering a range of operational, logistical, and service-related expenditures. These included Zipline’s central storage and aerial delivery of semen doses ($94,948), travel expenses for field teams ($23,040), training and workshops for Community Animal Health Workers ($16,015), and program management salaries ($12,000). Veterinary service fees accounted for $15,834, based on an average reimbursement of 3,000 RWF per insemination procedure. Altogether, these cost categories reflect the comprehensive resource inputs required to implement the intervention at scale, combining transport, technical services, and capacity-building components.

A total of 7,540 swine semen doses were procured for the program. Of these, 2,220 doses (29.44%) were sourced from the Rwanda Agriculture and Animal Resources Development Board (RAB) at a cost of 3,500 RWF per dose, while 5,320 doses (70.56%) were procured from private suppliers at 6,500 RWF per dose. The combined procurement cost, converted to USD at the prevailing exchange rate, totaled $29,312. Taken together, these cost components represent the total financial investment required to deliver the program at its observed scale (Table 3).

**Table 3:** Program costs.

Item	Cost	Source
Salaries	$12,000	PM from actuals in budget
Travel	$23,040	From actuals
Training/workshops	$16,015	From actuals
Flights	$94,948	14 USD per flight
Vets fees	$15,834	3000RWF per AI procedure
Swine semen doses	$29,312	From actuals
Total cost	$191,149	

#### 3.2.3 ROI

The swine artificial insemination program yielded substantial economic returns relative to its total implementation cost of $191,149. Based on direct income gains to participating farmers, the program generated an estimated $320,075 in revenue from piglet sales, resulting in a direct return on investment (ROI) of 67.5%. This corresponds to a benefit–cost ratio of 1.68, indicating that for every dollar invested, approximately $1.68 was returned in the form of direct, attributable income at the household level.

When accounting for broader economic effects, including indirect impacts on local service providers and induced effects arising from increased household spending, the total estimated economic impact increases to $2.3 million. This implies a total ROI of 1,106%, or $12.06 in overall economic value per dollar invested. These results suggest that, beyond improvements in farmer-level income, the intervention may contribute meaningfully to rural economic activity by strengthening livestock productivity and expanding access to reproductive inputs through aerial logistics.

## 4 Discussion

This evaluation of an aerial logistics model for swine AI services in Rwanda revealed substantial improvements in service delivery outcomes across multiple stakeholder groups. While veterinarians typically initiated semen orders and coordinated service delivery, CAHWs served as the frontline actors who directly interfaced with farmers and performed the insemination procedures. This cascade structure meant that while both groups participated in the AI ecosystem, CAHWs were uniquely positioned to observe outcomes firsthand and collect more accurate follow-up information. This distinction is critical to interpreting the results: although veterinarians reported stable AI success rates around 74.5–74.8%, CAHWs documented a statistically significant increase from 48.8% at baseline to 74.8% at endline. Because both groups were asked about outcomes using the same semen products, the observed difference likely reflects differences in data reliability rather than differences in performance. CAHWs’ proximity to the farms and their ongoing engagement with pig owners made them more dependable sources of outcome data, especially for tracking pregnancy success and diagnosing challenges on the ground. These findings highlight the importance of centering frontline perspectives in evaluating last-mile interventions.

Although this study did not include a parallel non-intervention control group, the observed AI success rates can be contextualized using published evidence from similar smallholder settings in Rwanda. Munyaneza (5), studying extended fresh semen use in rural smallholder pig farms in Northern Rwanda, reported an average of 1.89 inseminations per conception and a litter size of 7.94 ± 2.24 piglets. An average of 1.89 inseminations per conception corresponds to an approximate conception probability of 53% per insemination attempt, consistent with our baseline CAHW-reported success rate of 48.8%. The endline success rate of 74.8% observed in the present study approaches the upper range of fertility rates reported under improved management conditions in the literature, where conception rates of 80–90% have been documented in organized systems (10). These comparisons suggest that the magnitude of improvement observed in this study is biologically plausible and consistent with previously reported reproductive performance benchmarks in Rwanda and comparable contexts.

Beyond reproductive outcomes, the structured coordination between veterinarians and CAHWs represents an important implementation feature. By concentrating regulatory oversight and ordering functions at the veterinarian level while delegating routine field engagement to locally embedded CAHWs, the model leverages differential training costs and reduces unnecessary travel or duplication of services. This tiered approach, combined with centralized aerial logistics, may contribute to cost containment and improved service reach in rural contexts.

District-level variation among CAHWs further highlights the importance of local context in shaping AI outcomes. Success rates were significantly higher in Nyamagabe, Nyamasheke, and Rutsiro, even after controlling for individual-level characteristics. These differences may reflect localized differences in infrastructure, program implementation fidelity, or levels of institutional support, although further research would be needed to identify the exact mechanisms. Importantly, these findings underscore that technical capacity building efforts alone may not be sufficient to ensure uniform outcomes, and that implementation strategies should be tailored to regional contexts.

Another key insight from the study is the shifting landscape of perceived AI challenges. Across both veterinarians and CAHWs, technical or procedural errors decreased substantially over the course of the project, indicating improved skills and procedural adherence. Similarly, the proportion of failures attributed to expired semen or cold chain interruptions declined, suggesting that the drone-based delivery model successfully mitigated major logistical constraints that traditionally undermine AI outcomes. On the other hand, reports of heat detection challenges and animal health issues either remained stable or increased slightly, pointing to persistent constraints that may require distinct interventions such as improved diagnostic tools or broader animal health support.

Among farmers, the findings showed a 63.1% AI success rate and an average of 8 piglets per successful pregnancy. These results are particularly striking considering that the majority of sows had not previously undergone AI, and over half had never been pregnant. This supports the notion that the intervention successfully expanded access to reproductive technologies among first-time users and suggests a strong initial impact on productivity. However, it is important to acknowledge a key limitation: because only current users were surveyed, the study cannot account for individuals who may have discontinued AI use due to negative experiences. Additionally, the assumption that the number of doses corresponding to first-time users could be directly inferred from farmer reports may introduce uncertainty into some of the downstream economic estimates.

While the current study does not have direct comparators from previous drone-based AI interventions in Rwanda or similar contexts, its findings are consistent with broader evidence on the importance of timely, high-quality input delivery in enhancing smallholder productivity. The economic analysis demonstrates that even modest improvements in fertility and pregnancy rates can translate into substantial financial gains for rural households. When compared to other documented swine-sector initiatives in Rwanda, this program exhibits a notably higher short-term return. For instance, the *Rwanda Red Cross “Pass-on Piglet” Rotation* cited in the national ROI assessment, achieved an estimated 600% return on investment over 8 years, equivalent to an annualized return of approximately 28.6% (6). Similarly, the *PRISM (Project for Resilient & Inclusive Small Livestock Markets) - IFAD* program was projected to yield a long-term internal rate of return (IRR) between 19 and 26% over 20 years (7). By contrast, the aerial AI program delivered a direct ROI of 67.5% in a single year, indicating a substantially higher annual return. If maintained across multiple years, this suggests that drone-based logistics may offer a more capital-efficient model for improving livestock outcomes and rural incomes, particularly in hard-to-reach or underserved areas.

These findings carry several implications for policy and practice. First, interventions aimed at expanding reproductive technologies in rural contexts must address both logistical and human capacity barriers in tandem. Second, decentralized actors such as CAHWs can be highly effective delivery agents when adequately supported, suggesting that future investments should prioritize their training, tools, and integration into formal systems. Third, scalable technology platforms like Zipline’s aerial logistics model offer a promising mechanism to bridge the last-mile gap in perishable product delivery, with potential applications beyond AI, such as in veterinary pharmaceuticals or vaccines.

Limitations of the study include the reliance on self-reported success rates, potential recall bias, and the absence of a comparison group not exposed to drone deliveries. In addition, the regression models, while robust in identifying associations, cannot establish causal mechanisms behind the observed differences. Finally, as noted, the study did not capture non-users or dropouts, limiting insights into barriers to continued adoption.

Among the limitations of our economic analysis, given the methods applied, it is likely that our analysis underestimates the true economic impact of the program. First, we assume that the average litter size per successful insemination remains constant between the baseline and endline scenarios. However, evidence from the literature consistently indicates that improved swine semen quality, such as that achieved through better cold chain conditions, can lead to larger litter sizes.

Second, we assume that all farmers were using semen delivered by aerial logistics, when in reality the improved availability of AI services through aerial delivery could affect both the AI success rate and farmers’ willingness to use AI at all. Our endline survey indicates that 74% of the farmers served were first-time AI users, suggesting that at least some of the increase in AI use was driven by improved accessibility through aerial logistics. Finally, in calculating the economic impact, we use the market value of a piglet; however, for many subsistence-oriented households, swine production also functions as a critical safety net, providing animals that can be sold in times of catastrophic expenses or consumed during periods of food insecurity. Consequently, the real value of a piglet may exceed its market price, as it can represent survival in some cases. Moreover, since swine production is often managed by women, these income gains likely have important equity and redistributive effects that go beyond simple market transactions (8).

Although published research specifically examining drone-mediated delivery of reproductive inputs in livestock systems remains limited, there is growing evidence supporting the use of unmanned aerial vehicles for biological supply chain delivery in both animal and human health sectors. In Rwanda, for example, Griffith et al. (9) examined stakeholder perceptions of using drones to deliver Rift Valley Fever vaccines for livestock and highlighted the potential of aerial logistics to overcome cold-chain and geographic access constraints in rural settings. These findings align with broader operational experiences in medical supply chains across sub-Saharan Africa, where drones have been used to transport temperature-sensitive biological products to remote facilities.

Future research should explore longitudinal outcomes of AI use, including piglet survival, weight weaning, and long-term income changes. Additionally, qualitative work could deepen understanding of farmer and CAHW decision-making processes, barriers to adoption, and satisfaction with services. Comparative studies between districts or with regions not served by aerial logistics could help isolate the specific contribution of the drone delivery component.

## Data Availability

Anonymized data will be made available by the authors upon request and subject to assessment.
